# Abstracts and Gleanings

**Published:** 1885-08-20

**Authors:** 


### ABSTRACTS AND GLEANINGS.

**The Mind-Cure, New and Old.**—We have before us an address entitled, "Trust in the Infinite," which maintains that not only moral but physical infirmities are removable by trust in the Infinite; that they will and must disappear under certain states of mind.

The writer, however, ignores *means* similar to those which Christ himself used and recognized.

This is a powerful means of arousing mental dominion and imparting psychical tone, and thus of exciting the general tone of the organism in certain conditions, and revives an interest in the mind-cure of all diseases which was attributed to the gods.

A recent lecture by Prof. August C. Merriam, of Columbia College, New York, delivered March 19, at the New York Academy of Medicine, on the "Treatment of Patients in the Temples of Æsculapius," a synopsis of which we find in the New York Medical Record, is, in this connection, interesting reading:

"He gave a historical sketch of the methods of healing practiced in those temples, especially at Athens and Epidoris, and revealed by Grecian excavations begun March 16, 1881. The speaker pictured the deep and abiding belief which the ancients had in the anger or malice of the gods, as a *cause* of disease, and also in the marvellous and miraculous *cure* of disease by some divinity.

"The beginning of the art of medicine, in all times and among all peoples, was empiricism, based on the belief that all severe sicknesses were caused by the anger of some god or demon.

"The faith of the people in the power of the gods of Æsculapius to cure disease was unlimited, and was represented by the saying of Plato, who declared that 'we must not suppose that Æsculapius failed through ignorance, but that he deliberately withheld his power at times for the benefit of the race.'

"The writings of Solon, who mentions 'the laying-on-of-hands,' etc.; of Pythagoras, who was physician as well as philosopher; of Plato; of Aristophanes, and of other ancient Greeks, are all flavored with the belief in the influence and power of divinities.

"Galen, it seems, was nearly free from superstition, yet he refers to the partial-god Æsculapius. Three hundred and twenty temples of Æsculapius were known to have existed in antiquity: those of Athens and Epidoris having been built about the end of the fifth century before Christ.

"The speaker sprinkled his discourse with translations of inscriptions found upon slabs removed in recent excavations, some of which were highly amusing."

The mind-cure idea substitutes monotheism for polytheism, and credits the divinity within us with the power of expulsion of disease, while denying to any god or to the one God any influence in bringing on disease. "He healeth all diseases," think the mind-cure people, but chasteneth none with disease.—*Alienist and Neurologist.*

**The New Treatment for Typhoid Fever.**—The most recent abortive treatment for typhoid fever is suggestive of vigorous treatment for syphilis. Dr. Kalb, of Germany, is the originator of it, and he has proven its efficacy in more than a hundred cases. It is as follows: Rub into the skin 90 grains of mercurial ointment daily, for six days; make the first application to the abdomen: the second to the inside of one of the thighs, the third to the other thigh, the fourth to the abdomen again, and the fifth and sixth to the thighs as before. After each application, at least half an hour should be spent in rubbing it in; this must not be left to the patient, but should be done by an efficient nurse. It is preferable to make the inunction in the evening. At the same time he gives seven and one-half grains of calomel and three-quarters of a grain of opium internally every five or six hours, the opium being given to prevent cathartic action of the calomel. Alcohol is administered in full doses, but no other medicine is given.

On the second day the temperature falls one-half a degree or more, but the next day it rises again to its original height and remains at this point for seven days, and then falls to normal or nearly so, and does not rise again. But sometimes the temperature sinks to normal before the inunctions are completed; in which case they are to be continued just the same, for the temperature would be likely to rise again if discontinued. In a few days after the fall of the temperature the pulse falls to normal, and the patient becomes convalescent: the spleen, however, remains enlarged from ten to fourteen days.

Kalb has carefully compared these results with the results of other modes of treatment, which shows advantages in favor of the former. He has even been reproached by patients who had not been anointed, upon seeing the anointed patients up and about, while they themselves were still confined to bed. The treatment is not successful if begun after the first nine or ten days. About twenty per cent. of cases do not seem to be susceptible to this treatment, but the remaining eighty per cent. are cured in the above remarkable manner. Whether the typhoid poison is counteracted by the mercury as syphilitic poison seems to be, we cannot say; but the clinical facts stand out in bold relief. Just how much of the credit may be given to the mercury, and how much to the alcohol does not appear; perhaps the virtue lies in their combined action. We do not wish to be understood as endorsing this treatment, but we present it as being worthy of attention, to be proven or disproven by further experience.—*Medical World.*

**Malarial Hæmaturia.**—Dr. E. C. Rothrock, of Athens, Tex., says, in Medical World, it has been fully demonstrated that sulphate of quinine is the main factor in the cure of malarial diseases, yet there is one exception: malarial hæmaturia. At least in the acute stages quinine will do harm, as will the use of mercury and blisters. That such is the fact will be admitted by those who have had much to do with the disease. Why it is so is not my intention to discuss. The primary cause is malarial poison producing congestion of the liver, spleen and kidneys, embarrassment of their functions, also of the skin, intestines, etc. This is followed by a vitiated state of the blood ; a disintegration from accumulation in that fluid, of urea, uric acid, biliary matters, carbonic acid, etc., pernicious principles which are produced in the body in a state of health and should be eliminated by the emunctories as fast as formed. The deleterious effects of such excretions retained in the blood is well illustrated in the uremic form of this disease.

There is no doubt but what malarial hæmaturia is caused by minute organisms in the blood (call them what you choose,) a ferment as shown by a great tendency to metamorphosis—a decomposition of tissue. The disease will not yield (as other malarial diseases) to the ordinary anti-malarial treatment. The fever will not give way until an effective antiseptic treatment is instituted and the blood rendered aseptic. To meet indications, then, we give chlorate of potash, two and a half drachms, sweet spirit of nitre and water, of each one ounce ; give one drachm every two hours until the urine assumes a natural color. Also give hyposulphite of soda, ten grains, in plenty of water (two or three ounces) every three or four hours, or alternate with potash mixture. Eliminate hydro-carbon by the liver with black snake root (cimicifuga racemosa) and serpentaria virginia, thirty to sixty drops of each, every four hours until it acts; beware of stimulating the kidneys to excess; watch the action of the kidneys and if any sign of deficient secretion, use mild diuretics, as buchu, uva ursi, etc.; if hemorrhage is excessive, give rhus aromatica; control vomiting with cold water, sulphate of soda, bismuth sub-nit., ox. cerium, pepsin, etc.; cold, green tea is excellent. Hot foot-baths are good in some cases, and the entire body should be briskly rubbed with whisky and water, equal parts, with a tablespoonful of salt added to one quart; it should be used warm and continued for twenty or thirty minutes and repeated every three hours. It does great good in producing revulsion, stimulating the cutaneous circulation. A warm poultice applied to the epigastric region has frequently a good effect, and will sometimes abate a nausea that is always present. In twelve or twenty-four hours from commencement of treatment I give tincture of iron, ten, fifteen or twenty drops in water, three times a day. After the acute stage has abated, treat as ordinary malarial cases. This is the general treatment that I have used for years, and it has cured in every case.

**Induction of Premature Labor.**—Dr. T. Gaillard Thomas, of New York, writes as follows, regarding the induction of premature labor (Medical and Surgical Reporter, Feb. 14, 1885): The method of inducing premature labor which I now invariably adopt is very simple, and, at the same time, a perfectly efficient one. The patient is placed across the bed, with the buttocks resting near the edge, and under her is arranged a large piece of rubber or oil-cloth in such a way as to drain into a tube below on the floor. In this tube we put one or two gallons of water at a temperature of 98° F. The operator stands between the thighs of the patient, whose knees should be properly supported, and employing a syringe with a long nozzle, which is carried up as far into the cervical canal as it will go, he keeps a steady stream directly against the membranes. In the course of ten minutes the os will be the size of a silver half dollar, and when dilatation to this extent has been accomplished, he is to insert a gum catheter between the membranes and the uterine walls. The patient is then put in bed, and that is all.

This operation constitutes one of the greatest advances that have ever been made in the obstetric art, and it is certainly no mean triumph to be able thus to preserve a human life, which, without its aid, would have been inevitably lost. I can point to at least two dozen children in this city who by this means were saved from an untimely fate. When the infant has been delivered before full term, it should not be washed and otherwise treated in the ordinary manner of nurses, but should be carefully wrapped in warm cotton and allowed to remain in it, the temperature of the room in the meanwhile being brought up to nearly one hundred degrees.—*Maryland Med. Journal.*

**Iodine and Pyridine in the Treatment of Asthma.**—The alkaloid termed pyridine ($C_5H_5N$) is a colorless, volatile liquid, of a very penetrating odor, miscible with water, and forming with the mineral acids, salts which are very soluble. It is obtained by the dry distillation of various organic substances, such as Dippel's animal oil, certain alkaloids (including cinchonine, quinine, morphine, and atropin), and coal tar. It is found also in the condensed products of tobacco smoke and in nicotine itself. M. Germain See lately read an interesting paper before Academie des sciences (Rev. med.) on the use of pyridine in the treatment of asthma. He declares that iodine is the greatest curative agent in this affection, whatever form it may take, and that pyridine is the best palliative for use during the attacks. It causes a decided and immediate diminution of the feeling of oppression, so that the breathing becomes calm, while the action of the heart preserves its regularity as well as its force. After about an hour there is in many cases an irresistible desire to go to sleep, but no stupor or any approach to anæesthesia, although the reflex excitability is diminished. The remedy should be given by inhalation, a fluid-drachm or more being poured on to a napkin in a close room. The inhalation should be continued for twenty or thirty minutes, and should be repeated three times a day. After two or three inhalations, auscultation will show great improvement of the physical signs. A few persons seem to have their susceptibility to the action of the drug impaired at the end of a week or ten days, and then it is well to begin with the iodine treatment.—*N. Y. Medical Journal.*

**Death Caused by a Purgative in a Case of Intestinal Occlusion.**—An interesting case of death following the administration of a dose of castor-oil has been reported by M. Nicaise to the Clinical Society of Paris. The patient, a man of forty-five years of age, had a narrowing of the small intestine following a strangulated inguinal hernia, which was operated on in 1875.

There were recent digestive troubles, vomiting, and intestinal colic, but the stools were regular and appetite good. Following a dose of one ounce of oil were symptoms of obstruction of the bowel, with rapid collapse and sign of failure of vital powers. Acting upon the idea of the existence of a stricture of the small intestine following the hernia, laparatomy was performed, but no constricting band was found. Enterotomy was then performed, and the contents of the distended intestine allowed to escape. There was no prietonitis and no effusion. A pouch-like dilatation of the small intestine was found just above the constricted portion, the communication between the two portions being not at the bottom, but to one side, in such a way that the increased muscular contractions, and excessive secretion, under the influence of the purgative, had compressed the opening into the constricted portion and produced complete occlusion.

This case demonstrates the danger of purgatives in intestinal obstruction, and the necessity of prompt resort to operative measures for relief. The utility of washing out the stomach in such a case appears very evident.—*Medical Times.*

**An Almost Painless Mode of Introducing a Catheter where there is Hyperæsthetic Condition of the Urethra.**—Dr. Stamps, in Medical and Surgical Reporter, says: This consists in introducing the nozzle of an ordinary male urethral syringe (previously filled with water as warm as the patient can bear) into a soft catheter, and injecting the water slowly as the catheter is gently passed along the urethral canal.

If the bladder is not entered by the time the first syringeful is exhausted, refill the syringe and proceed as before.

You will find that the water will regurgitate between the catheter and urethal wall till the catheter has reached the prostatic portion, thus lessening the danger of injecting too much water into the bladder.

My experience with this method is very limited, yet it has proved very successful in the few cases I have had, and as I have never seen an account of it in any medical works or journals, I submit it to the profession for a trial and report of its success. The following is a case showing its value:

J., æt. 20, male, of nervous temperament, suffering from cystitis (the result of a gonorrhœa of several months' standing), which was attended with all the suffering characteristic of this trouble, and with an abundance of pus, mucous, blood, and heavy deposit of the phosphates in his urine.

He had to micturate every twenty or thirty minutes, both day and night, and had been so for two months, so I concluded to wash out his bladder; but on attempting to introduce the catheter (which was a soft rubber one), I found the pain so intense as to cause spasm of the sphincter vesicæ, thus rendering all efforts at entering the bladder fruitless. The thought then occurred to me, that, by injecting warm water and putting the urethra slightly on the stretch, I could pass the catheter; so I procured the syringe and again started the catheter, keeping a steady flow of water playing on the urethra, and very much to his comfort and my satisfaction, I succeeded in entering his bladder without causing, as he stated, the least pain.

**Overdose of Jaborandi.**—Mrs. C., aged 39, was delivered of her first child seven weeks ago. The supply of milk, which had been copious, began to subside two weeks ago, and had almost entirely disappeared. Ordered a teaspoonful, three times a day, of a mixture of equal parts of fluid extract of jarborandi (P. D. & Co.) and glycerin. On getting the medicine, the patient, instead of measuring the dose, placed the medicine to her mouth and took a swallow. I was telephoned for two hours later. Found the patient in a severe rigor, shaking as if in an attack of ague, her clothes being as completely saturated with perspiration as if she had been plunged into the river, and the saliva flowing freely from her mouth. Pulse, 130, and feeble ; temperature, 98°. Gave a hypodermic injection of morphine, gr. 1/6, and atropine, gr. 1-120, and administered per os a half ounce of brandy. Under this treatment there was a rapid improvement in the distressing and somewhat alarming symptoms. On examination of the bottle, I found that 5 drachms of the mixture, containing ʒijss of the jaborandi, had been taken. There was a marked stimulation of the flow of milk. The jarborandi was ordered continued in the doses originally prescribed, and with the most satisfactory results.—*Dr. J. J. Mulheron, in Medical Age.*

**Non-vesicating Croton-Oil.**—An important discovery seems to have been made by Mr. Harold Senier, of the London Chemical Society, to judge from an abstract given in a recent number of the Lancet of a paper read by him at a meeting of the Pharmaceutical Society. It amounts to nothing less than that Croton-oil may be separated into two different oils by the action of alcohol, one of which is irritating but not purgative, and the other purgative but not irritating. When alcohol of the specific gravity of 0.794 to 0.800 is added to Croton-oil in the proportion of seven or eight more volumes to six, the oil separates into two parts—one of them (the vesicating oil) dissolves in the alcohol, and remains soluble in alcohol in all proportions ; the other (the purgative oil) separates, and is then found to have become insoluble in any proportion of alcohol. This insoluble oil is said to be a safe and pleasant purgative, free from any undesirable action, in doses of one-tenth to one-half a minim, in the form of pills made with magnesium carbonate and extract of henbane as excipients.—*N. Y. Med. Jour.*

**Statistics of General Paralysis.**—J. Luys (L'Encephale, 1884, No. 6—Documents Statistiques pour servis a l'Etude des Conditions Pathogeniques de la Paralysie Generale), basing his conclusions on 140 cases of general paralysis, found that the average age in men is forty-three, in women forty years. In the higher classes of society there is a decided predominance of male cases, in the lower the percentage is about even. The scarcity of progeny in paralytics is very striking, 27 (34 per cent.) of 81 marriages proving sterile ; 53 fertile marriages produced only 80 children, consequently only 1.5 each. The children of paralytics are generally of irregular development, bodily as well as mentally. Statistics prove that the mental state of the mother exerts the greatest influence on the male progeny. In nineteen out of twenty cases the brothers or sisters of paralytics show peculiarities in their manners, although there are many exceptions. Luys comes to the conclusion that general paralysis is not a disease coming from without, like scarlet fever or variola, but that it is the last link in the long chain of preparatory morbid conditions.—*Alienist and Neurologist.*

**How to Diagnose Gonorrhœa in the Female.**—The difficulty of differentiating a specific vaginitis from a simple catarrhal inflammation of the vagina, has probably worried most of our readers. A mistake in diagnosis in these cases is also a matter of very considerable importance. The happiness of a home may hang on the issue. It becomes the physician in such a case to hew to the line, let the chips fall where they may ; but he must be particularly careful that none of them fall on his own toes. There has, up to the present time, been no pathognomonic sign which might serve as a guide in such a perplexity. At a recent meeting of the Paris Obstetrical and Gynæcological Society, however, Martineau suggested one which may answer the purpose. The pus of the specific vaginitis is said to be always acid, while in the simple variety it is alkaline. A little piece of litimus paper, therefore, will tell the story. The importance of this discovery cannot well be over-estimated. Both on account of social and medico-legal reasons, its importance is very great.—*Medical Age.*

**The Action of Warm Water Upon the Gravid Uterus.**—Auvard (Bull. gen. de Therap.) reports the result of a number of observations made for the purpose of determining whether warm vaginal injections could be used with safety during pregnancy, and whether they had influence upon the course of labor. He decides that water at a temperature 48° C.(=118.4° F.) does not cause uterine contractions, if injected slowly and gently into the vagina. Kiwisch's results with injections of warm water for the purpose of inducing premature labor, must, therefore, the author thinks, have been due either to the violence of the treatment, or to the fact that other means were employed in addition. If these injections are practiced every half hour during the first stage of labor, the os dilates rapidly and with less pain than when they are not used.—*Cin. Med. News.*

**Glycerine as a Tænicide.**—Dr. G. A. McCallum, of Dunville, Ontario, writes to the British Medical Journal, of June 13, that glycerine is the most efficient tænicide that he has yet seen. In some cases it may be necessary to follow the glycerine with a gentle purgative. He has found that when the head of the worm is placed in glycerine, it dies almost immediately. This fact is especially important in treating cases of tænia in children.—*Journal Am. Med. Association.*

